# Geographic Variation and Factors Associated with Female Genital Mutilation among Reproductive Age Women in Ethiopia: A National Population Based Survey

**DOI:** 10.1371/journal.pone.0145329

**Published:** 2016-01-07

**Authors:** Tesfaye Setegn, Yihunie Lakew, Kebede Deribe

**Affiliations:** 1 Bahir Dar University, College of Medicine and Health Sciences; School of Public Health, Department of Reproductive Health, Bahir Dar University, Bahir Dar, Ethiopia; 2 Ethiopian Public Health Associations, Addis Ababa, Ethiopia; 3 Brighton and Sussex Medical School, Falmer, Brighton, United Kingdom and Addis Ababa University School of Public Health, Addis Ababa, Ethiopia; Universität Bochum, GERMANY

## Abstract

**Background:**

Female genital mutilation (FGM) is a common traditional practice in developing nations including Ethiopia. It poses complex and serious long-term health risks for women and girls and can lead to death. In Ethiopia, the geographic distribution and factors associated with FGM practices are poorly understood. Therefore, we assessed the spatial distribution and factors associated with FGM among reproductive age women in the country.

**Method:**

We used population based national representative surveys. Data from two (2000 and 2005) Ethiopian demographic and health surveys (EDHS) were used in this analysis. Briefly, EDHS used a stratified, two-stage cluster sampling design. A total of 15,367 (from EDHS 2000) and 14,070 (from EDHS 2005) women of reproductive age (15–49 years) were included in the analysis. Three outcome variables were used (prevalence of FGM among women, prevalence of FGM among daughters and support for the continuation of FGM). The data were weighted and descriptive statistics (percentage change), bivariate and multivariable logistic regression analyses were carried out. Multicollinearity of variables was assessed using variance inflation factors (VIF) with a reference value of 10 before interpreting the final output. The geographic variation and clustering of weighted FGM prevalence were analyzed and visualized on maps using ArcGIS. Z-scores were used to assess the statistical difference of geographic clustering of FGM prevalence spots.

**Result:**

The trend of FGM weighted prevalence has been decreasing. Being wealthy, Muslim and in higher age categories are associated with increased odds of FGM among women. Similarly, daughters from Muslim women have increased odds of experiencing FGM. Women in the higher age categories have increased odds of having daughters who experience FGM. The odds of FGM among daughters decrease with increased maternal education. Mass media exposure, being wealthy and higher paternal and maternal education are associated with decreased odds of women’s support of FGM continuation. FGM prevalence and geographic clustering showed variation across regions in Ethiopia.

**Conclusion:**

Individual, economic, socio-demographic, religious and cultural factors played major roles in the existing practice and continuation of FGM. The significant geographic clustering of FGM was observed across regions in Ethiopia. Therefore, targeted and integrated interventions involving religious leaders in high FGM prevalence spot clusters and addressing the socio-economic and geographic inequalities are recommended to eliminate FGM.

## Introduction

Female Genital Mutilation (FGM) is a partial or total removal of the external female genitalia for non-medical reasons[1, 2]. FGM is a worldwide problem affecting almost all ethnic groups[3]. Globally, in 2008, there were an estimated 100–140 million women and girls who underwent FGM. The practice is primarily performed in Africa where more than 28 countries and more than 3 million girls are at risk of experiencing FGM[3, 4]. In East Africa; Somalia (98%), Djibouti (93%), Eritrea (89%) and Ethiopia (74%) have the highest FGM prevalence[1]. Although there is limited documentation regarding the magnitude and factors associated with FGM due to mysteries surrounding its practice, there is a more recent shift in attitudes toward addressing this traditional practice [4, 5].

Currently, the World Health Organization (WHO) classifies four types of FGM: Type I, *clitoridectomy*, involving partial or total removal of the clitoris and/or the prepuce; Type II, *excision*, involving partial or total removal of the clitoris and the labia minora, with or without excision of the labia majora; Type III, *infibulation*, involving narrowing of the vaginal orifice with creation of a covering seal by cutting and appositioning the labia minora and/or the labia majora, with or without excision of the clitoris; and Type IV, *other*, involving all other harmful procedures to the female genitalia for non-medical purposes, such as pricking, piercing, incising, scraping, and cauterization[1, 2].

In Ethiopia, FGM is widespread across the majority of regions and ethnic groups, with Type I and II having the highest national prevalence [6]. The national estimated prevalence of FGM among girls and women (age 15–49 years) is 79.9% [7] and 74.3% [8], respectively. The prevalence is estimated to be highest in Afar (91.6%), Somali (97.3%) and Dire Dawa (92.3%) [8] regions.

FGM has varying socio-cultural meanings, degrees of practice and support for its continuation or discontinuation. The practice of FGM is maintained due to social and family pressure to adhere to tradition and the meaning passed on from generation to generation. Even women support the continuation of FGM for these reasons, leaving other girls and women to indefensibly suffer the consequences [9, 10]. Depending on the type, FGM poses complex socio-cultural and serious sexual and reproductive health risks for women and girls[11]. FGM threatens the health and wellbeing of women, causing hemorrhage, infection, prolonged labour, pain during sexual intercourse, gynecological and urogynaecological problems [12–16]. It can be linked to increased long term complications and maternal deaths [4, 9].

Underscoring the socio-economic and health consequences of FGM, the Ethiopian government has shown high levels of political commitment to end FGM. The government has been implementing various interventions and FGM has been declining across Ethiopia [17]. However, there has been little evidence of the factors associated with FGM and its geographic distribution. This lack of evidence is detrimental to designing interventions for the areas with highest FGM prevalence and indicates a need to study the geographic variation of FGM and associated socio-economic factors. Hence, identifying geographic areas with higher prevalence spots and clusters is an important turning point for the rapid reduction and elimination of FGM through better targeted geographic and population interventions. Therefore, this study identifies the spatial clustering and socio-economic and demographic factors associated with FGM among reproductive age women in Ethiopia.

## Methods

### Study setting and design

Administratively, Ethiopia is divided into nine regional states and two city administrations. Each regional state is divided into zones, zones into administrative units called *weredas (*districts), and weredas are further subdivided into the lowest administrative units called *kebele*s [18].

This analysis was based on the 2000 and 2005 Ethiopian Demographic and Health Surveys (EDHS). Since the EDHS 2011 data did not contain FGM questions, it was not considered for this analysis. The two surveys followed an international DHS methodology and were conducted in five year intervals. The surveys were designed to provide population and health indicators at national (urban and rural) and regional levels. The 2000 and 2005 EDHS samples were selected using two-stage stratified cluster sampling. A total of 29,437 women (15,367 from 2000 and 14,070 from 2005 EDHSs) in the reproductive age group (15–49 years) were included. The response rates were 95.6% and 97.8% for the 2000 and 2005 EDHS respectively. The detailed methodologies of the two surveys can be found elsewhere [12, 19, 20].

### Instrument and data extraction

The surveys used standard questionnaires developed by MEASURE DHS. The questionnaires were translated into the three major languages of Ethiopia. The women’s questionnaire was used to generate data related to FGM from all women aged 15–49 years[21]. The 2000 and 2005 EDHS datasets were downloaded from the Measure DHS website (http://www.dhs.program.com) in SPSS format with permission. Since the FGM data was not collected in the EDHS 2011, the analysis was limited to EDHS 2000 and EDHS 2005. After understanding the detailed datasets and coding, further data recoding was done. All potential social determinants and FGM indicator variables were extracted from the EDHS datasets. The socio-economic, demographic and geographic coordinate datasets were merged and analyzed.

### Measurements

There were three outcome variables which were measured and coded as "Yes = 1” or “No = 0". Women having experienced FGM was measured as a ‘Yes’ response for the question “Have you *ever been circumcised*?” Daughters having experienced FGM was measured as a ‘Yes’ response for the question “*Is your daughter circumcised*?” Women in support of FGM continuation was measured as a ‘Yes’ response for the question “*Do you support FGM to continue*?*”* Independent variables presumed to affect the practice of FGM for women and their daughters and their support of FGM continuation, such as exposure to mass media, wealth status, occupation, religion, age, women and men educational status, were measured based on the respondents’ reports. Geographic region and residential areas of the women (urban, rural) were included in the analysis.

### Data analysis

Data was analyzed using STATA version 11 software. The two data sets were weighted (to compensate for unequal probability of selection among geographic strata) using the “svy” command [19]. Descriptive statistics (weighted prevalence and percentage changes), bivariate and multivariable logistic regression analyses were carried out to determine the factors associated with FGM. As recommended by Hosmer and Lemeshow, statistically significant variables at p-value <0.25 during bivariate logistic regression analysis were taken for the multivariable logistic regression model[22]. This p-value cutoff point is important in order to retain variables that will have potential effect during multivariable analysis. All tests were two-sided and a p-value <0.05 was considered as the cut-off point for statistically significant association in the multivariable model. Both unadjusted and adjusted odds ratios were calculated with a 95% confidence interval (CI). Multicollinearity between independent variables was assessed by using variance inflation factors (VIF) (reference value of 10) before interpreting the final output.

The percentage change was calculated as an end year value (percentage) of a specific indicator minus the beginning year value (percentage) divided by the value (percent) in the beginning year and multiplied by 100, or $\mathrm{PC}=\frac{\left(\mathrm{Percentage values in}\;2005\;\mathrm{EDHS}-\mathrm{Percentage values in}\;2000\;\mathrm{EDHS}\right)}{\mathrm{Percentage values of}\;2000\;\mathrm{EDHS}}*100$

The weighted prevalence data was exported to ArcGIS to visualize estimations. The geographically clustered FGM prevalence rates were identified and defined as “high” or “low” FGM prevalence spots were located on maps. The geographic variation of clustered FGM prevalence spots were assessed using z-score values. A positive z-score indicates high prevalence clustering and a negative z-score indicates low clustering of FGM prevalence.

### Ethical consideration

The study is based on secondary data analysis of EDHS. The study was approved by the ORC Macro Research Ethics Committee, Ethiopian Public Health Institute (EPHI) and Ethiopian Science and Technology Agency. Prior to the actual interviews, informed consent was obtained from the participants, their guardian or household heads. Hence, for this analysis all the identifications related to the participants were removed. The GIS data were obtained through direct review and approval from MEASURE DHS.

## Results

### Trends of FGM and mothers’ experiences

The prevalence of FGM among women was 79.9% (95%CI: 79.26, 80.53) in 2000 and 74.3% (95%CI: 73.57, 75.02) in 2005. During the 2000 EDHS, the prevalence rate of FGM among rural and urban populations was relatively similar. However, in the EDHS 2005, the prevalence of FGM among urban and rural population was 68.5% and 75.5% respectively. The prevalence rate of FGM also showed regional variation. The highest prevalence was documented in Somali region both in 2000 and 2005 EDHS (***Table 1***).

**Table 1:** Trends and Geographic Variation of Female Genital Mutilation among Reproductive age women in Ethiopia; 2000–2005, Ethiopia.

Geographic locations	EDHS 2000		EDHS 2005		Percentage changes
	Weighted total number of women	Weighted prevalence of circumcision (95%CI)	Weighted total number of women	Weighted prevalence of circumcision (95%CI)	
**Region**					
Tigray	969	35.7(32.73,38.76)	919	29.3(26.40,32.28)	-17.9
Afar	178	98.6(96.34,99.81)	146	91.6(86.45,95.47)	-7.1
Amhara	3820	79.7(78.41,80.96)	3480	68.6(67.03,70.12)	-13.9
Oromia	5937	89.8(89.00,90.54)	5010	87.2(86.26,88.11)	-2.9
Somali	175	99.7(97.21,99.97)	486	97.3(95.58,98.50)	-2.4
Benishangul-Gumz	160	73.7(66.52,80.13)	124	67.6(59.14,75.52)	-8.3
SNNPR	3285	73.5(71.96,74.97)	2995	71.0(69.34,72.59)	-3.4
Gambella	40	42.9(27.97,58.08)	44	27.1(15.70,41.76)	-36.8
Harari	41	94.3(84.81,99.17)	39	85.1(70.72,93.52)	-9.8
Addis Ababa	684	79.9(76.85,82.84)	756	65.7(62.30,69.06)	-17.8
Dire Dawa	79	95.1(88.24,98.37)	69	92.3(84.67,97.30)	-2.9
**Residence**					
Urban	2791	79.8(78.27,81.25)	2499	68.5(66.67,70.31)	-14.2
Rural	12576	79.9(79.19,80.59)	11570	75.5(74.71,76.27)	-5.5
Total	15367	79.9(79.26,80.53)	14069	74.3(73.57,75.02)	-7.0

### Daughters’ experience of FGM and geographic variations

Based on the 2000 EDHS, the national prevalence of FGM among daughters was 47.8% (95%CI from 46.72 to 48.87). However, this figure decreased to 37.7% (95%CI: 36.64, 38.77) during the 2005 EDHS. The percentage change between EDHS 2000 and 2005 among urban daughters was 41.9% and 30.0%, respectively. The FGM prevalence among daughters also showed a statistically significant geographic variation; where Afar and Gambella regions had the highest and lowest FGM prevalence (***Table 2***).

**Table 2:** Daughters' experience of FGM and geographic variation in Ethiopia, EDHS 2000–2005.

Geographic location	EDHS 2000		EDHS 2005		Percentage changes
	Weighted total number of daughters circumcised	Weighted prevalence of daughters circumcised (95%CI)	Weighted total number of daughters circumcised	Weighted prevalence of daughters circumcised (95%CI)	
**Region**					
Tigray	555	29.6(25.86,33.45)	524	30.2(26.34,34.19)	2.0
Afar	95	93.2(87.33,97.40)	82	85.1(76.45,91.82)	-8.7
Amhara	2241	70.5(68.59,72.37)	2,014	56.8(54.63,58.96)	-19.4
Oromia	3159	42.4(40.67,44.12)	2,873	34.9(33.18,36.67)	-17.7
Somali	101	57.4(47.64,66.79)	323	28.1(23.47,33.27)	-51.0
Benishangul-Gumz	87	56.6(45.78,66.45)	74	49.3(37.42,59.98)	-12.9
SNNPR	1761	32.3(30.16,34.52)	1733	23.5(21.54,25.53)	-27.2
Gambella	20	24.1(9.79,47.02)	25	11.0(3.15,29.28)	-54.4
Harari	19	44.6(21.83,64.63)	17	27.1(11.66,53.68)	-39.2
Addis Ababa	227	39.6(33.43,46.12)	223	25.1(19.75,31.12)	-36.6
Dire Dawa	33	39.6(23.45,56.63)	32	34.3(19.58,51.88)	-13.4
**Residence**					
Urban	1124	41.9(39.04,44.81)	914	30.0(27.07,33.01)	-28.4
Rural	7174	48.7(47.55,49.86)	7007	38.7(37.57,39.85)	-20.5
Total	8298	47.8(46.72,48.87)	7920	37.7(36.64,38.77)	-21.1

### Women’s support of FGM continuation

The women’s support of FGM continuation showed significant decrease from 65.0% (95%CI: 64.21, 65.78) in 2000 EDHS to 31.4% (95%CI: 30.60, 32.20). During the 2000 EDHS, 72.9% (95%CI: 64.21, 65.78) of rural women were supporters of FGM continuation. Women from different regions showed statistically significant differences in supporting FGM continuation. In this regard, women from Somali and Afar regions were the major supporters of FGM continuation with 77.3% and 76.8%, respectively. Women from Addis Ababa were the least supportive of FGM continuation in both EDHS 2000 and 2005 (16.3% and 5.6%). The SNNP region showed the highest change and reduction of women’s support of FGM continuation (***Table 3***).

**Table 3:** Women's support for FGM continuation and geographic variation in Ethiopia, EDHS 2000–2005.

Geographic location	EDHS 2000		EDHS 2005		Percentage changes
	Weighted total number of women	Weighted prevalence to be continued (95%CI)	Weighted total number of women	Weighted prevalence to be continued (95%CI)	
**Region**					
Tigray	694	35.4(31.95,39.06)	762	21.6(18.84,24.68)	-39.0
Afar	177	76.8(70.19,82.61)	143	65.7(57.67,73.17)	-14.5
Amhara	3380	68.1(66.52,69.66)	3094	39.1(37.40,40.84)	-42.6
Oromia	5835	70.9(69.72,72.05)	4866	29.8(28.53,31.10)	-58.0
Somali	175	77.3(70.48,82.91)	476	74.5(70.52,78.34)	-3.6
Benishangul- Gumz	141	60.9(52.76,68.78)	99	40.1(31.08,50.28)	-34.2
SNNPR	2892	67.9(66.19,69.59)	2597	26.0(24.33,27.70)	-61.7
Gambella	22	47.8(29.78,70.22)	20	21.0(6.70,41.49)	-56.1
Harari	40	51.6(37.12,67.53)	39	21.6(10.01,35.26)	-58.1
Addis Ababa	679	16.3(13.71,19.27)	753	5.6(4.10,7.40)	-65.6
Dire Dawa	79	45.6(34.84,56.61)	69	13.8(7.60,24.31)	-69.7
**Residence**					
Urban	2704	32.0(30.25,33.77)	2445	10.4(9.23,11.65)	-67.5
Rural	11410	72.9(72.08,73.71)	10473	36.3(35.39,37.23)	-50.2
Total	14114	65.0(64.21,65.78)	12918	31.4(30.60,32.20)	-51.7

### Factors associated with women’s experience of FGM

Women in the richest and richer wealth index categories had higher odds of having experienced FGM as compared to women in the poorest category. Muslim women had 3 times higher odds of having experienced FGM as compared to Orthodox women (p<0.0001). Women in the higher age categories had higher odds of experiencing FGM as compared to young women (15–19 years) (***Table 4***).

**Table 4:** Factors associated with women’s experience of FGM among reproductive age women in Ethiopia, EDHS 2011.

*Variables*	*Unadjusted OR (95%CI)*	*Adjusted OR (95%CI)*
**Wealth index**		
Poorest	Reference	Reference
Poorer	1.2(0.94,1.44)	1.2(0.90,1.50)
Middle	1.1(0.91,1.42)	1.2(0.92,1.59)
Richer	1.3(1.02,1.65)	1.4(1.03,1.86)*
Richest	1.2(0.94,1.60)	1.6(1.09,2.28)*
**Religion**		
Orthodox	Reference	Reference
Catholic	0.8(0.47,1.26)	0.8 (0.39,1.76)
Protestant	0.8(0.63,0.93)*	0.9 (0.69,1.23)
Muslim	2.7(2.16,3.26)*	3.1(2.36,4.21)***
Traditional	0.7(0.34,1.45)	0. 5(0.19,1.10)
Others^$^	0.7(0.35,1.26)	0.8(0.30,1.30)
**Age of women (years)**		
15–19	Reference	Reference
20–24	1.9(1.65,2.22)	1.2(0.94,1.62)
25–29	2.5(2.15,2.94)	1.5(1.12,1.92)**
30–34	2.9(2.43,3.50)	1.6(1.23,2.18)**
35–39	4.3(3.55,5.26)	2.3(1.74,3.14)***
40–44	5.0(3.96,6.29)	2.9(2.12,4.05)***
45–49	4.9(3.88,6.23)	2.8(1.99,3.85)***

^$^ Jehovah, other unclassified

*P<0.05

**p<0.001

***p<0.0001

### Factors associated with daughters’ experience of FGM

Daughters of richer women, Muslim mothers and mothers aged ≥25 years had higher odds of having experienced FGM. Daughters from richer women showed 40% higher odds of having experienced FGM as compared to daughters of the poorest women. Compared with Orthodox women, Muslim women were two times more likely to have their daughter circumcised. Daughters of women in the higher age categories showed higher odds of having experienced FGM as compared with daughters of young women aged 15–19 years. On the other hand, daughters of Protestant women had lower odds of having experienced FGM. Higher levels of maternal education were associated with 80% lower odds of FGM experience for daughters **(*Table 5***).

**Table 5:** Factors associated with daughters’ circumcision among reproductive age women in Ethiopia, EDHS 2011.

*Variables*	*Unadjusted OR (95%CI)*	*Adjusted OR (95%CI)*
Wealth index		
Poorest	Reference	Reference
Poorer	0.9(0.73,1.113)	1.0(0.81,1.31)
Middle	1.0(0.76,1.19)	1.2(0.92,1.54)
Richer	1.1(0.84,1.34)	1.4(1.06,1.83)*
Richest	0.8(0.63,1.06)	1.3(0.90,1.0)
Women occupation		
Not working	Reference	Reference
Professional/technical/managerial	0.4(0.23,0.78)*	0.7(0.31,1.69)
Clerical	0.4(0.13,0.98)*	0.8(0.25,2.25)
Sales	1.1(0.88,1.29)	1.0(0.81,1.28)
Agriculture-employee	1.3(1.06,1.60)*	1.2(0.93,1.47)
Services	0.6(0.11,3.50)	2.0(0.31,12.21)
Skilled manual	1.5(0.99,2.24)	1.3(0.78,2.04)
Unskilled manual	0.7(0.430,1.09)	0.6(0.335,0.97)*
Religion		
Orthodox	Reference	Reference
Catholic	1.0(0.45,1.75)	0.7(0.33,1.49)
Protestant	0.5(0.37,0.63)***	0.6(0.40,0.75)***
Muslim	1.8(1.46,2.25)***	2.0(1.52,2.55)***
Traditional	0.5(0.23,1.11)	0.4(0.15,0.96)*
Others^@^	0.8(0.32,1.79)	0.60(0.22,1.60)
Age		
15–19	Reference	Reference
20–24	1.1(0.65,1.84)	1.1(0.64,1.79)
25–29	1.9(1.17,3.11)*	1.9(1.15,3.08)*
30–34	4.3(2.64,7.08)***	4.4(2.66,7.17)***
35–39	9.3(5.70,15.24)***	9.2(5.62,15.16)***
40–44	20.5(12.38,33.90)***	19.4(11.64,32.23)***
45–49	32.6(19.60,54.37)***	28.4(16.93,47.59)***
Maternal education		
No formal education	Reference	Reference
Primary	0.4(0.35,0.52)***	0.7(0.58,0.91)*
Secondary	0.2(0.14,0.26)***	0.3(0.23,0.50)***
Higher	0.1 (0.07,0.27)***	0.2(0.07,0.50)*

^**@**^ Jehovah, other unclassified

*P<0.05

***p<0.0001

### Factors associated with women’s support of FGM

In this analysis, being a rural resident and Muslim were factors positively associated with women’s support of FGM. Rural women were two times more likely to support FGM continuation as compared with urban women. Similarly, Muslim women were about two times more likely to support FGM continuation as compared with Orthodox women. Women exposed to media had 20% lower odds of supporting FGM continuation as compared with their counterparts. Women in the middle, rich and richest wealth index categories had 30%, 20% and 30% lower odds, respectively, of supporting FGM continuation as compared with the poorest women. Although a higher level of paternal education was not statistically associated with FGM support, primary and secondary paternal education showed 20% and 30% lower odds of support for FGM continuation, respectively. All levels of women’s formal education was associated with lower odds of supporting FGM continuation (***Table 6***).

**Table 6:** Factors associated with women’s support of female genital mutilation continuation; among reproductive age women in Ethiopia; 2000–2005.

Variable	Unadjusted OR with 95%	Adjusted OR with 95%
**Exposed to mass media**		
No	Reference	Reference
Yes	0.5(0.48,0.59)***	0.8(0.73,0.95)*
**Wealth index**		
Poorest	Reference	Reference
Poor	0.9(0.73,1.01)	1.0(0.80,1.14)
Middle	0.6 (0.53,0.75)***	0.7(0.60,0.87)*
Rich	0.6(0.48,0.69)***	0.8(0.63,0.94)*
Richest	0.3(0.21,0.33)***	0.7(0.51,0.86)*
**Occupation**		
Not working	Reference	Reference
Professional/technical/managerial	0.2(0.09,0.36)***	0.9(0.33,2.53)
Clerical	0.6(0.29,1.36)	1.7(0.45,6.09)
Sales	1.0(0.84,1.141)	1.0(0.84,1.22)
Agriculture—employee	1.4(1.15,1.59)***	1.1(0.92,1.30)
Services	0.4(0.05,3.06)	1.3(0.14,11.43)
Skilled manual	0. 7(0.46,0.99)*	0.6(0.37,0.93)*
Unskilled manual	1.2(0.82,1.69)	0.9(0.59,1.41)
**Religion**		
Orthodox	Reference	Reference
Catholic	1.0(0.61,1.65)	0.7(0.38,1.34)
Protestant	0.8(0.65,1.00)	0.7(0.58,0.95)*
Muslim	2.3(1.96,2.80)***	1.8(1.51,2.21)***
Traditional	1.4(0.74,2.69)	0.8(0.39,1.62)
Others^#^	1.0(0.51,1.78)	0.7(0.35,1.45)
**Partner education**		
No formal education	Reference	Reference
Primary	0.6(0.56,0.73)***	0.8(0.68,0.91)*
Secondary	0.4(0.29,0.43)***	0.7(0.55,0.88)*
Higher	0.1(0.05,0.18)***	0.4(0.20,0.88)
**Residence**		
Urban	Reference	Reference
Rural	7.4(5.57,9.85)***	2.1(1.45,2.99)***
**Maternal education**		
No formal education	Reference	Reference
Primary	0.4(0.39,0.50)***	0.76(0.64,0.90)*
Secondary	0.12(0.09,0.16)***	0.29(0.20,0.43)***
Higher	0.06(0.03,0.14)***	0.09(0.02,0.45)*

^#^ Jehovah, other unclassified

*P<0.05

***p<0.0001

### Geographic variation and spatial clustering of FGM

**Fig 1:**
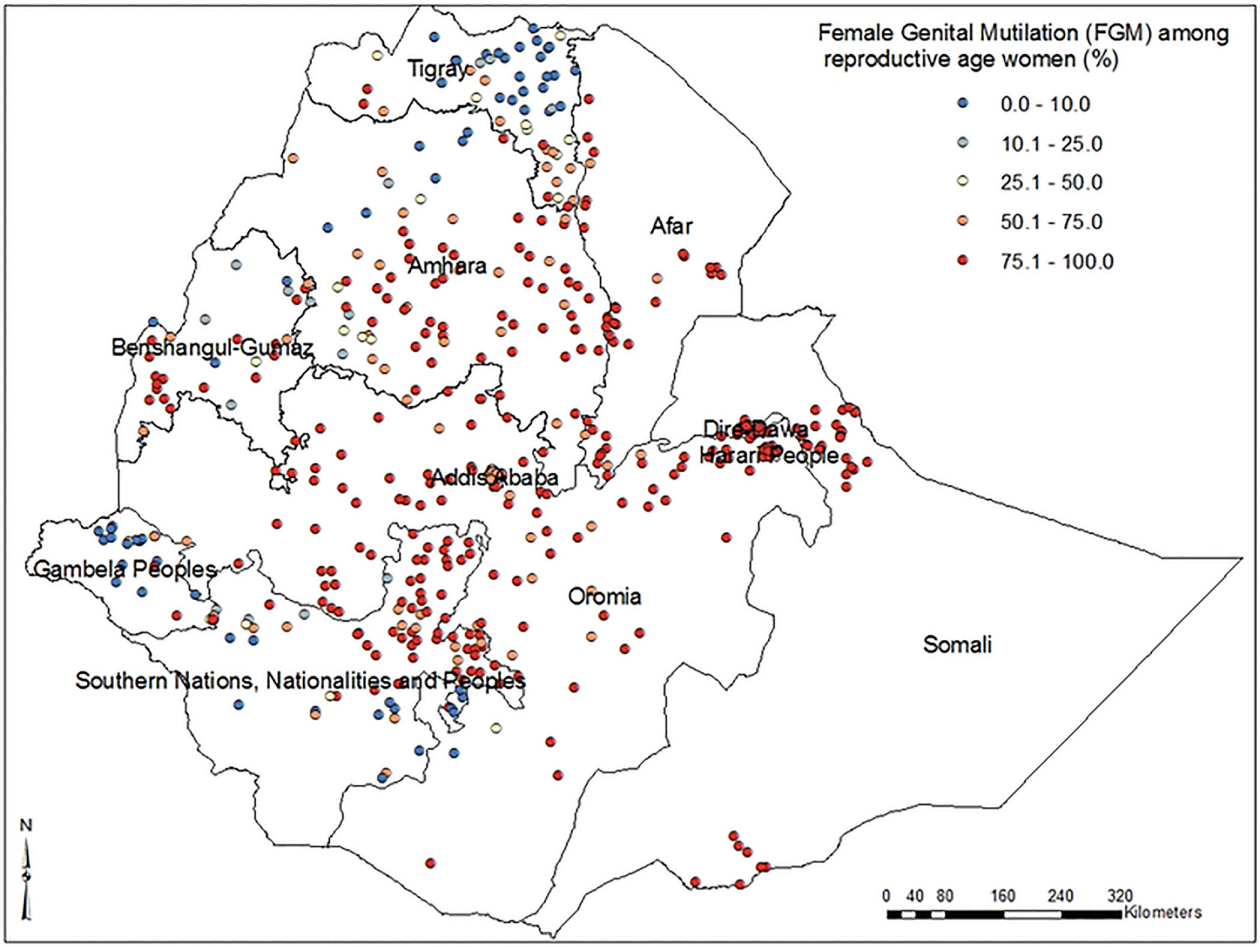
Map of regional distribution of female genital mutilation prevalence among reproductive age women in Ethiopia; 2005.

**Fig 2:**
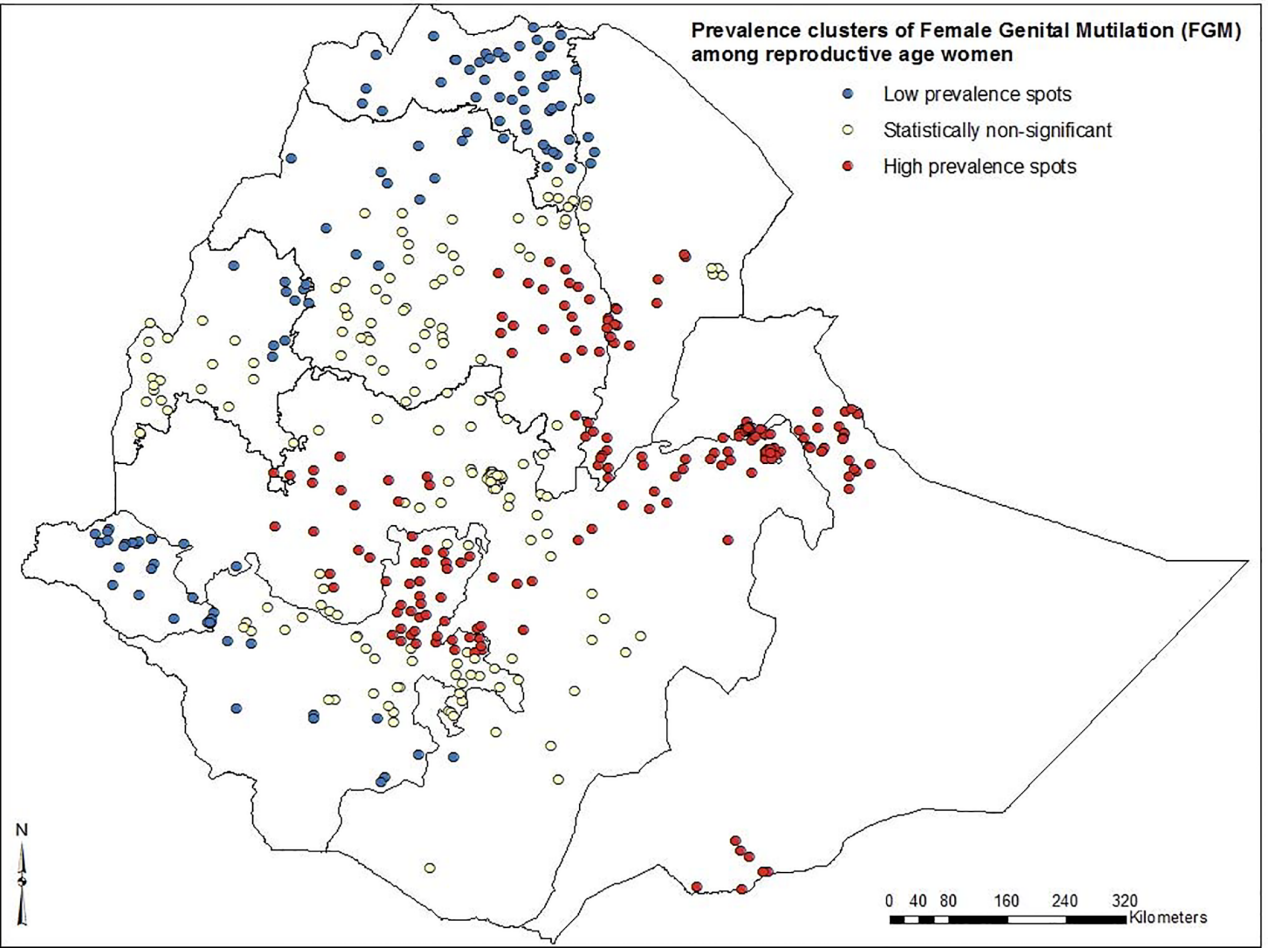
Spatial clustering of high and low prevalence spots of female genital mutilation among reproductive age women in Ethiopia; 2005.

In this study, low FGM prevalence spots were found in the eastern part of Tigray, north Amhara, east Benishangul-Gumz and Gambella regions. High FGM prevalence spots were observed in central and eastern parts of Amhara region, Afar, Dire-Dawa, east and west Oromia, Somali and northern parts of SNNP regions (***Fig 1)***. The spatial distribution of FGM showed significant geographic variations among regions in Ethiopia (***Fig 2***).

**Fig 3:**
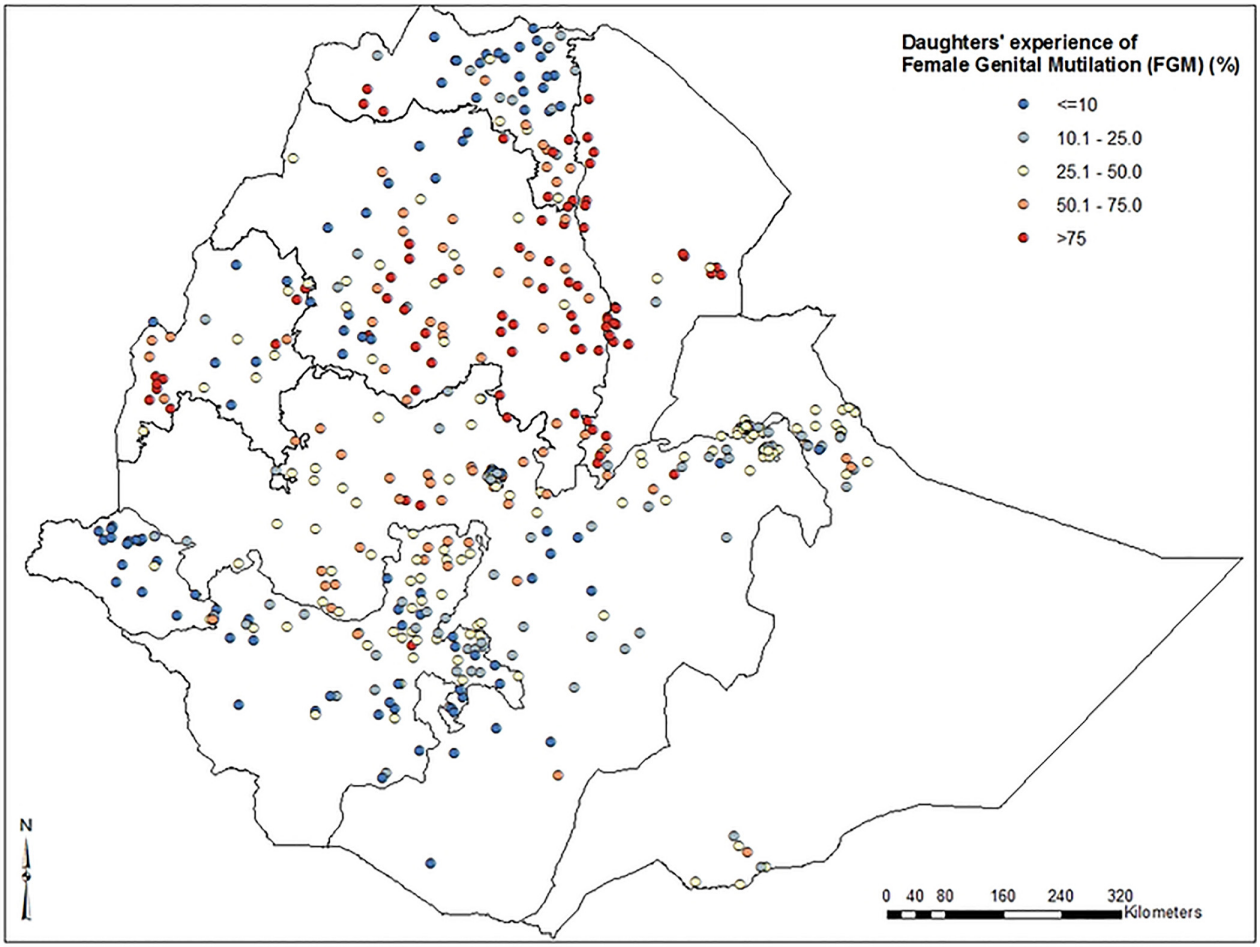
Map of prevalence distribution of female genital mutilation among daughters in Ethiopia; 2005.

**Fig 4:**
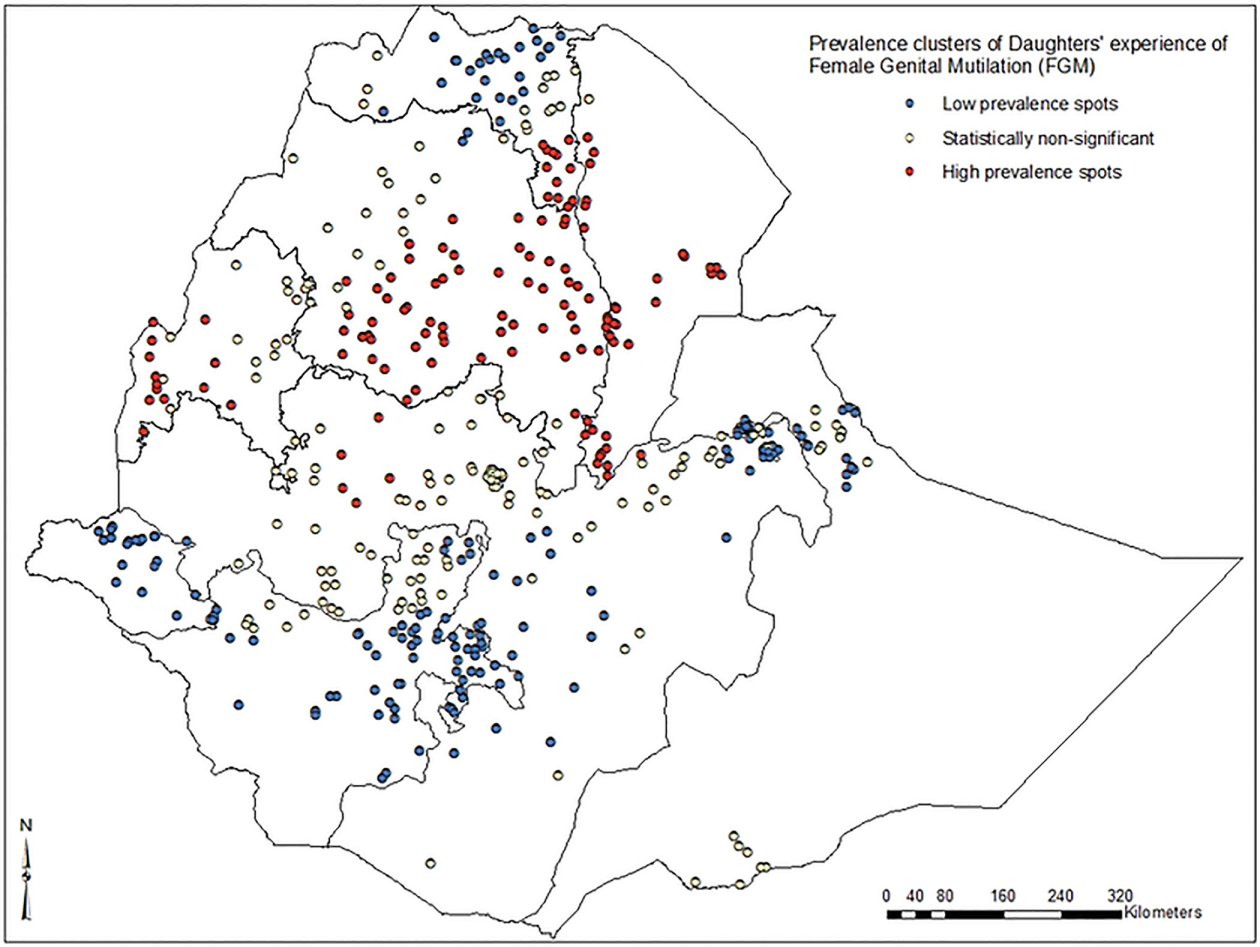
Spatial clustering of high and low prevalence spots of female genital mutilation among daughters in Ethiopia; 2005.

Low prevalence of FGM among daughters was identified in the eastern part of Tigray, north and eastern parts of Amhara, Gambella, eastern part of Oromia and northern parts of SNNP regions. High prevalence of FGM (>75%) among daughters was identified in southern Tigray, central and southern parts of Amhara, north-western and southern parts of Afar and some parts of Benishangul-Gumz regions (***Fig 3***). Further spatial clustering analysis of daughters’ experience of FGM showed a statistically significant variation among regions in Ethiopia. High prevalence of daughters’ FGM experience spots were clustered around southern Tigray, central and southern parts of Amhara, and north western and southern parts of Afar regions (***Fig 4***).

**Fig 5:**
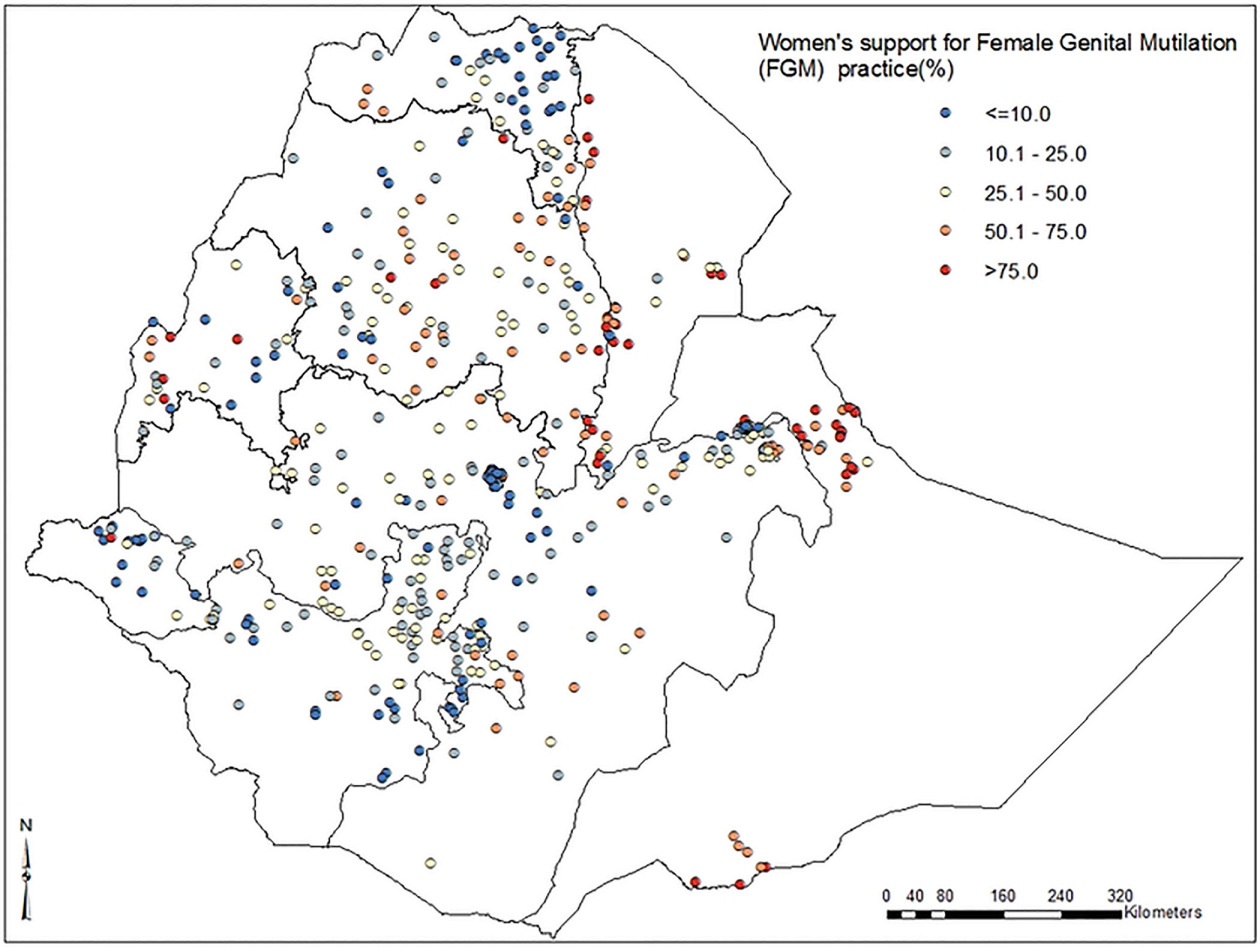
Map of prevalence distribution of women's support for female genital mutilation continuation among women in Ethiopia; 2005.

In our study, a high proportion of women’s support for FGM (>75%) continuation was recorded in some central and south east parts of Amhara, north west and south west parts of Afar, and north and south east parts of Somali regions (***Fig 5***).

## Discussion

In this study we used two nationally representative population based surveys from Ethiopia. Our results showed a decreasing trend of FGM prevalence over time and significant variation across geographic and socioeconomic groups. Similarly, a study conducted in Kenya reported geographic variation of FGM prevalence [23]. Studies conducted in Jigjiga [24], Bale Zone[14] and Kersa district[25] in Ethiopia showed that 90%, 78.5% and 94% of women had undergone FGM respectively. Similar, studies in Burkina Faso[26] and Gambia[16] indicated that 77% and 75.6% of women had experienced FGM. As with other studies [23, 26–31] our study identified that age of mothers, religion, place of residence (urban versus rural), household wealth index, maternal education and ethnicity were factors associated with maternal and/or daughters’ FGM experience.

In our study, higher order wealth index, older age of women and being Muslim were factors associated with increased FGM risk. Other studies in Ethiopia have indicated similar factors associated with FGM practice [24, 32]. Studies conducted in Kenya[23] and Sierra Leone [5] have identified that religion, wealth status and perceived benefits of FGM were independent predictors of FGM practice. In our study, daughters with mothers who were Muslim, of higher age categories and illiterate had increased odds of having experienced FGM.

The relationship between Muslim religion and FGM practice and support found in high clustered regions such as Afar, Somali and Dire-Dawa might be due to the fact that these regions are majorly populated by Muslims [12, 19, 20, 24] and the Muslim community perceives that FGM (“Sunna type”) is an important tradition[24] and religious requirement for Muslim women [5, 32]. This indicates a need for developing faith-based interventions that involve Muslim religious leaders, change social expectations and perceived religious requirements in order to reduce FGM. Moreover, majority of women in high FGM prevalence clusters are rural residents, less educated and illiterate [12, 19, 20]. They are more likely to conform to traditional practices, including FGM. This may indicate a need to target FGM reduction interventions at the high clustered rural areas and intensify educational enrollment of women and girls.

Different studies [5, 23, 28] have shown that complex socio-cultural factors and women’s support play major roles in the continuation of FGM [5]. In our study, although women with better wealth status were less likely to support the continuation of FGM, better socio-economic status (wealth) is a factor associated with FGM practice. This might be associated due to the decision making power of women in relation to their wealth. Evidence has shown that well-to-do women were the decision makers on FGM practice. Similarly, mass media exposure, better paternal and maternal education, and socio-economic status were associated with decreased odds of women’s support of FGM continuation. This might be due to the overall community-driven change associated with media exposure [5] and education [5, 23, 33, 34]. Wider community mobilization and education are associated with the overall empowerment of women and their capacity to fight against harmful traditional practices such as FGM. Therefore, women’s education is a development priority in order to foster fast and longstanding behavioral change to eradicate FGM.

### Strengths and limitations

The use of nationally representative data and identified factors related to FGM and support of its continuation, assessment of geographic distribution and identification of FGM high spot clusters are the strengths of this study. Limitation of this study is that it did not assess community related factors such as norms, beliefs and cultural values which influence FGM practice. This study was also unable to investigate the varying decision making roles of males versus women in the practice and continuation of FGM. Additionally, small counts for some variable categories may create difficulty in interpreting specifics related to that category. Information bias, potential residual confounders and minimal level of covariate interactions might limit the results. Therefore, any scientific interpretation or conclusion based on this study should consider these limitations.

### Implication for intervention

Our analysis showed important socioeconomic, demographic factors and geographic variation in FGM practice in Ethiopia. Although FGM practice decreased between 2000 and 2005 significantly, the battle against FGM practice is far from over. The identified factors associated with FGM can be used to reach out to segments of the population that are still practicing FGM. Support to FGM continuation is evident high FGM prevalent areas. The geographic variation and spatial clustering will inform targeted interventions to areas that are hotspots of FGM practice. The efforts made in these high prevalence areas will contribute to significant reduction of the overall national FGM prevalence. FGM continues to be a public health challenge and requires a multi-disciplinary approach. Further research in the area is also required, including multilevel modeling of the individual, social and structural factors affecting FGM practice. Detailed comparison of high and low spot areas in terms of differing legal enforcement, available interventions and cultural and societal factors are needed. It is time to scale up effective local interventions to the national scale.

## Conclusion

In this study, the practice and support of FGM among reproductive age women was found to have decreased over time. However, the reduction was more significant in urban versus rural areas, where the majority of the nation’s population is living. Although Ethiopia has made important progress toward the elimination of FGM, the achievements so far have fallen short of the goal. The practice of FGM is deep-rooted, declining slowly and needs targeted intervention. This suggests the need for comprehensive and intensified community based efforts in the rural and high FGM practice clustered regions of Ethiopia. This study also concluded that FGM is associated with socio-economic factors (i.e. wealth status and education), demographics and religious practices. This suggests the need for integrated societal development interventions, such as involving religious leaders in women’s empowerment and community mobilization interventions, targeted at the high FGM clustered rural areas and regions.
